# Depletion of *Fkbp5* Protects Against the Rapid Decline in Ovarian Reserve Induced by Prenatal Stress in Female Offspring of Wild-Type Mice

**DOI:** 10.3390/ijms26062471

**Published:** 2025-03-10

**Authors:** Monica Moore, Busra Cetinkaya-Un, Papri Sarkar, Umit A. Kayisli, Nihan Semerci-Gunay, Michael Teng, Charles J. Lockwood, Ozlem Guzeloglu-Kayisli

**Affiliations:** 1 Department of Obstetrics and Gynecology, Morsani College of Medicine, University of South Florida, Tampa, FL 33602, USA; monicamoore@usf.edu (M.M.); busracetinkayaun@usf.edu (B.C.-U.); paprisarkar@usf.edu (P.S.); uakayisli@usf.edu (U.A.K.); nsemerci@usf.edu (N.S.-G.); cjlockwood@usf.edu (C.J.L.); 2 Department of Internal Medicine, Morsani College of Medicine, University of South Florida, Tampa, FL 33612, USA; mteng@usf.edu

**Keywords:** FKBP51, ovarian reserve, prenatal stress, female reproduction, aging

## Abstract

Prenatal stress (PNS) impairs offspring ovarian development by exerting negative long-term effects on postnatal ovarian function and folliculogenesis. FKBP51 is a stress-responsive protein that inhibits glucocorticoid and progesterone receptors. We hypothesize that FKBP51 contributes to impaired ovarian development and folliculogenesis induced by PNS. Timed-pregnant *Fkbp5*^+/+^ (wild-type) and *Fkbp5*^−/−^ (knockout) mice were randomly assigned to either the undisturbed (nonstress) or PNS group, with exposure to maternal restraint stress from embryonic days 8 to 18. Ovaries from the offspring were harvested and stained, and follicles were counted according to their stages. Ovarian expressions of FKBP51 were evaluated by immunohistochemistry and *Fkbp5* and steroidogenic enzymes were evaluated by qPCR. Compared to controls, *Fkbp5*^+/+^ PNS offspring had increased peripubertal primordial follicle atresia and fewer total follicles in the adult and middle-aged groups. In adult *Fkbp5*^+/+^ offspring, PNS elevated FKBP51 levels in granulosa cells of primary to tertiary follicles. Our results suggest that PNS administration increased FKBP51 levels, depleted the ovarian reserve, and dysregulated ovarian steroid synthesis. However, these PNS effects were tolerated in *Fkbp5*^−/−^ mice, supporting the conclusion that FKBP51 contributes to reduced ovarian reserve induced by PNS.

## 1. Introduction

Stress significantly impacts psychological, physical, and physiological states by disrupting homeostasis [1]. Stress-associated disorders are more common in women, who also experience faster and more prolonged stress responses than men [2]. This is due to the close interaction between the female reproductive system and the hypothalamic–pituitary–adrenal (HPA) [3] and –ovarian (HPO) axes [4]. By activating these axes, stress inhibits female reproductive function by impairing ovarian function and follicle development, as well as reducing oocyte number and/or quality [5]. Specifically, stress-induced activation of the HPA axis prompts the hypothalamus to release corticotropin-releasing hormone (CRH), stimulating the pituitary gland to secrete adrenocorticotropic hormone (ACTH), which triggers the adrenal cortex to produce and release glucocorticoids (cortisol in humans and primarily corticosterone in rodents) [6]. Elevated glucocorticoid levels negatively affect the hypothalamus and pituitary gland by inhibiting the secretion of gonadotropin-releasing hormone (GnRH). This, in turn, reduces the release of luteinizing hormone (LH) and follicle-stimulating hormone (FSH) [7], which are crucial for ovarian functions, including follicular growth, ovulation, and the production of steroid hormones estrogen and progesterone (P4) [8]. Elevated glucocorticoid levels also increase the formation of reactive oxygen species (ROS) in the ovary [9,10,11], which induce apoptosis in oocytes and granulosa cells, promote meiotic errors in the oocyte, and trigger oocyte fragmentation, resulting in decreased oocyte quality and quantity [12,13,14,15].

A growing body of research indicates that maternal stress and prenatal stress (PNS), defined as stressful life events during gestation, significantly impact pregnancy outcomes and negatively affect both the mother and child’s health, including functions of reproductive organs throughout the lifespan [16]. However, the exact mechanisms by which PNS causes later reproductive dysfunction in female offspring remain unknown. We previously demonstrated that maternal restraint stress activated the maternal HPA axis, as evidenced by significant increases in serum corticosterone and cortisol levels in both *Fkbp5*^+/+^ and *Fkbp5*^−/−^ dams [17]. Similarly, studies in sheep [18] and nonhuman primates [19] have shown that prenatal stress increases maternal cortisol levels during early to mid-gestation, which in turn elevates fetal cortisol circulation and suppresses fetal ACTH production. As pregnancy progresses toward parturition, elevated levels of placental 11β-hydroxysteroid dehydrogenase 2 (11β-HSD-2) begin to inactivate cortisol as it enters fetal circulation, acting as an incomplete barrier. This reduction in fetal circulating cortisol is thought to subsequently activate the fetal HPA axis, which plays a crucial role in promoting the growth and development of the fetal adrenal glands [20].

Previous studies in mice and rats have shown that PNS, by causing aberrant HPA axis development through an unclear mechanism [21], alters the expression of genes involved in ovarian steroidogenesis and decreases systemic estradiol [22,23] and P4 levels [24] while increasing corticosterone levels in adult offspring [25]. PNS in rats also impairs the estrous cycle, resulting in prolonged estrous cycles in the offspring [24]. Additionally, adult female offspring prenatally exposed to dexamethasone (DEX) as a model for PNS display delayed puberty, reduced ovarian volume, and decreased numbers of follicles in rats [26,27], as well as decreased germ cell density, as seen in human fetal ovaries after exposure to DEX [28].

FK506-binding protein 51 (FKBP51), a 51-kDa immunophilin, is a co-chaperone protein that interacts with heat shock proteins 90, 70, and p23, as well as steroid receptors glucocorticoid receptor (GR) and progesterone receptor (PR). It plays a role in immunoregulation as well as protein folding and trafficking [29]. FKBP51 levels are robustly upregulated by glucocorticoid and, to a lesser extent, by progestins [30,31,32,33]. However, elevated FKBP51 levels reduce P4 and glucocorticoid responses by inhibiting ligand binding and nuclear translocation, thereby negatively regulating GR and PR transcriptional activity [29]. Given FKBP51’s critical role in steroid receptor signaling, it is not surprising that altered FKBP51 levels are linked to many psychiatric and endocrine-related diseases, including depression, anxiety, and post-traumatic stress disorders, cancers, and metabolic and immune-related diseases [34].

Moreover, our lab recently identified the following: (1) significantly elevated FKBP51 levels in the nuclei of decidualized endometrial stromal cells from laboring versus non-laboring term placentas [32] and (2) increased FKBP51 levels in the decidua of patients with idiopathic preterm birth (PTB) versus gestational age-matched controls [17]. Consistent with the role of FKBP51 in labor, we also found that compared to *Fkbp5* wild-type (^+/+^) mice, *Fkbp5* knockout (^−/−^) mice display extended gestation and are resistant to maternal stress-induced PTB [17]. These results suggest FKBP51 is a potential mediator of PNS.

As a key modulator of the stress response, FKBP51 levels are affected by *FKBP5* gene polymorphisms, epigenetic modifications, aging, and early-life stress [35,36,37]. *Fkbp5*-deficient mice exhibit resistance to stress due to enhanced HPA axis feedback, altered stress signaling pathways, and improved stress resilience [38]. Stress effects on female fertility include a decline in ovarian reserve, which is crucial for female fertility, since females are born with a finite number of oocytes, and the quantity and quality of oocytes declines with age [39,40].

A growing body of evidence suggests that prenatal stress (PNS) is associated with accelerated ovarian aging [41,42]; however, the mechanisms by which PNS affects ovarian aging are not yet well understood. Wang et al. found that prenatal exposure to famine in Chinese women was linked to an increased risk of premature ovarian failure and early menopause [43]. Animal models have also explored various prenatal stressors and their effects on reproductive aging. For example, prenatal malnutrition in rats led to reduced ovarian vascularity, a sign of ovarian aging [44]. Prenatal immunological stress was associated with early ovarian senescence [45], while chronic prenatal hypoxia resulted in several indicators of accelerated ovarian aging in adult offspring, including a decreased reserve of primordial follicles, increased oxidative stress, impaired DNA repair mechanisms, and shortened ovarian telomere lengths [46]. The decline in reproductive ability with age is related to a decreased ovarian reserve and damage to remaining oocytes, such as reduced mitochondrial activity and increased aneuploidy [47,48,49]. Unlike somatic cells, oocytes’ telomeres shorten with age due to damage from ROS [48]. Follicular fluid and oocytes from older individuals exhibit altered antioxidant profiles, impairing their ability to scavenge ROS [9,50]. Additionally, significant stress levels have been shown to produce follicular phenotypes similar to those observed in aged ovaries, suggesting that stress may contribute to accelerated ovarian aging [11,13]. Therefore, in the current study, we hypothesize that PNS negatively impacts female ovarian function, leading to reduced ovarian reserve in female offspring. Additionally, we propose that PNS-mediated and/or age-related effects are mitigated in *Fkbp5*^−/−^ mice, potentially extending ovarian reproductive lifespan, as FKBP51 levels increase with age [35,51]. Thus, to test this hypothesis, we analyzed the effect of PNS on ovarian reserve by examining female offspring subjected to maternal restraint stress in both wild-type and *Fkbp5*-deficient mouse models.

## 2. Results

### 2.1. PNS Administration Affects the Follicular Reserve in Adult Fkbp5^+/+^ Mice

**Figure 1:**
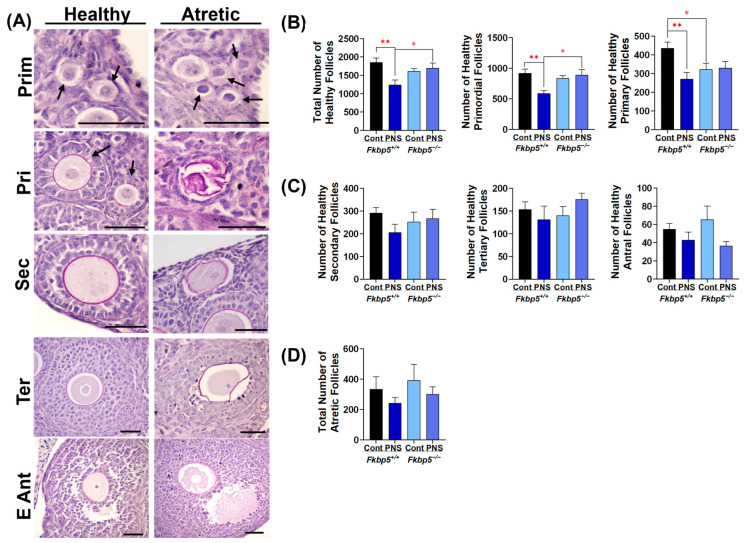
PNS administration reduced follicular numbers in *Fkbp5*^+/+^ adult offspring. (**A**) Representative image of PAS-stained healthy and atretic follicles at various developmental stages: primordial (Prim), primary (Pri), secondary (Sec), tertiary (Ter), and early antral (E Ant). Scale bars = 20 µm; (**B**) the total number of healthy primordial and primary follicles; (**C**) numbers of healthy growing secondary, tertiary, and antral follicles; (**D**) total number of atretic follicles. Bars represent mean ± SEM; * *p* < 0.05 or ** *p* < 0.01; *n* = 6/group. Arrows indicate primordial follicles.

To investigate the effect of PNS and its association with *Fkbp5* on the ovarian reserve, we first assessed follicle development in the ovaries of unstimulated 2-month-old mature *Fkbp5*^+/+^ and *Fkbp5*^−/−^ mice, subjected to either a prenatally unrestrained (physiologic) state (control) or a prenatally (maternal restraint) stressed (PNS) state. Follicles at all developmental stages were present in the ovaries of both *Fkbp5*^+/+^ and *Fkbp5*^−/−^ mice under both control and PNS conditions (Figure 1A). No significant difference was detected for the total number of healthy follicles between *Fkbp5*^+/+^ versus *Fkbp5*^−/−^ control groups (1851.7 ± 122.9 versus 1614.6 ± 70.7; *p* = 0.1, Figure 1B). However, significantly reduced numbers of healthy follicles were detected between PNS-induced *Fkbp5*^+/+^ mice (1240.4 ± 133.2) versus *Fkbp5*^+/+^ control mice (1851.7 ± 122.9; *p* < 0.01), but not between PNS-induced *Fkbp5*^−/−^ mice versus *Fkbp5*^−/−^ control mice (1698.3 ± 138.4 versus 1614.6 ± 70.7; *p* = 0.6, Figure 1B). Additionally, the total number of healthy follicles was significantly lower in PNS-induced *Fkbp5*^+/+^ versus PNS-induced *Fkbp5*^−/−^ mice (1240.4 ± 133.2 versus 1698.3 ± 70.7; *p* < 0.05, Figure 1B).

Similarly, PNS-induced *Fkbp5*^+/+^ mice also displayed significantly lower numbers of primordial and primary follicles compared to *Fkbp5*^+/+^ control mice (590 ± 45.6 versus 917.1 ± 67.5, *p* < 0.01; 270 ± 35.9 versus 435 ± 33, *p* < 0.01, respectively, Figure 1B), but no differences were noted between PNS-induced *Fkbp5*^−/−^ mice versus *Fkbp5*^−/−^ control mice (888.3 ± 90.3 versus. 835.4 ± 42.4, *p* = 0.6; 330 ± 34.4 versus 321.2 ± 33.5, *p* = 0.9, respectively, Figure 1B). Significantly fewer primordial follicles were reported in PNS-induced *Fkbp5*^+/+^ mice compared to PNS-induced *Fkbp5*^−/−^ mice (*p* < 0.05), yet no difference in primary follicles was seen (*p* = 0.3). However, the number of primary follicles was lower in *Fkbp5*^−/−^ control mice compared to *Fkbp5*^+/+^ control mice (*p* < 0.05). There were no significant differences in the number of growing follicles, including secondary, tertiary, and antral ones, among groups (Figure 1C).

To investigate whether the PNS-induced reduction in the follicle number is linked to increased follicular atresia, we counted atretic follicles in both *Fkbp5*^+/+^ and *Fkbp5*^−/−^ mice under control and PNS conditions. The number of atretic follicles at each follicular stage did not differ among groups (Figure 1D). These results are also supported by active caspase-3 immunostaining showing no differences among groups (Figure S1A,B). Active caspase-3 immunostaining was primarily detected in granulosa cells of growing follicles, but not in primordial or primary follicles (Figure S1A).

### 2.2. PNS Increases FKBP51 Expression in Granulosa Cells in Adult Offspring Ovary

**Figure 2:**
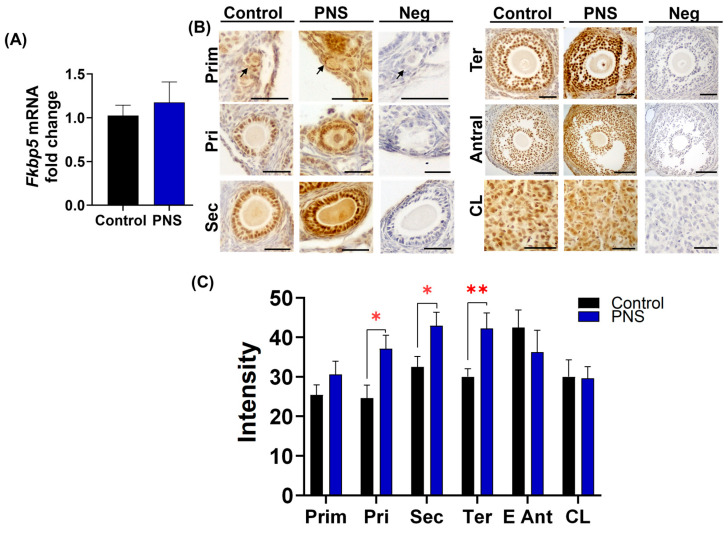
Increased FKBP51 immunostaining in granulosa cells of ovaries from offspring exposed to PNS. (**A**) Expression of *Fkbp5* mRNA levels in whole ovaries from control and PNS-exposed *Fkbp5*^+/+^ offspring. Mean ± SEM; 1.02 ± 0.1 versus 1.17 ± 0.2, *n* = 6/group; independent samples *t*-test *p* = 0.6. (**B**) Representative images showing FKBP51 immunoreactivity (brown) in granulosa and luteal cells from *Fkbp5*^+/+^ control and PNS mice. Arrows indicate primordial follicles. Scale bars = 20 µm. (**C**) Quantification of FKBP51 immunostaining intensity in every follicular stage and the corpus luteum. Bars represent mean ± SEM; *n* = 6 mice/group, compared to PNS versus control, primordial (Prim) (30.6 ± 3.3 versus 25.4 ± 2.5; *p* = 0.2); primary (Pri) (37.1 ± 3.4 versus 24.7 ± 3.3; * *p* < 0.05); secondary (Sec) (42.9 ± 3.4 versus 32.5 ± 2.7; * *p* < 0.05); tertiary (Ter) (42.3 ± 3.9 versus 30.0 ± 2.0; ** *p* < 0.01); early antral (E Ant) (36.3 ± 5.5 versus 42.5 ± 4.4; *p* = 0.3); corpus luteum (CL) (29.6 ± 2.9 versus 30.0 ± 4.3; *p* = 0.9).

As shown in Figure 1B, PNS-induced reduction in primordial and primary follicle numbers did not happen in *Fkbp5*^−/−^ mice. Thus, we first examined ovarian *Fkbp5* expression in control and PNS-induced *Fkbp5*^+/+^ mice. Analysis of qPCR results revealed that ovarian *Fkbp5* mRNA levels were similar in control versus PNS-induced *Fkbp5*^+/+^ mice (1.0 ± 0.1 versus 1.2 ± 0.2; *p* = 0.6, Figure 2A). Subsequently, ovarian FKBP51 levels were analyzed by immunohistochemistry using anti-goat FKBP51 antibody, which revealed immunoreactivity in several cell types, including oocytes, granulosa, cumulus, theca, luteal, and endothelial cells. FKBP51 was weakly expressed in oocytes throughout all follicular stages, from primordial to antral follicles, but was intensely expressed in granulosa cells across all follicle stages and luteal cells in the corpus luteum (Figure 2B). Next, we compared follicle stage-specific immunostaining of FKBP51 in control versus PNS-induced mice. FKBP51 immunoreactivity in granulosa cells of primordial follicles was similar in control and PNS-induced *Fkbp5*^+/+^ mice (Figure 2B). However, stronger FKBP51 immunoreactivity was observed in granulosa cells of primary, secondary, and tertiary follicles in PNS-induced versus control groups of *Fkbp5*^+/+^ mice (Figure 2B). Semi-quantitative analysis of signal intensity confirmed significantly higher FKBP51 levels in primary (37.1 ± 3.4 versus 24.7 ± 3.3; *p* < 0.05), secondary (42.9 ± 3.4 versus 32.5 ± 2.7; *p* < 0.05), and tertiary follicles (42.3 ± 3.9 versus 30.0 ± 2.0, *p* < 0.01) in the PNS versus control groups of *Fkbp5*^+/+^ mice (Figure 2C). No difference in FKBP51 intensity was seen in primordial follicles in the PNS versus control groups of *Fkbp5*^+/+^ mice (30.6 ± 3.3 versus 25.4 ± 2.5, *p* = 0.2). Additionally, no significant difference was observed in FKBP51 intensity in granulosa cells of antral follicles (*p* = 0.3) or in luteal cells (*p* = 0.9) of corpus luteum between the PNS and control groups of *Fkbp5*^+/+^ mice (Figure 2C).

### 2.3. PNS Reduces Hsd11b2 Levels in Adult Fkbp5^+/+^ Mice

**Figure 3:**
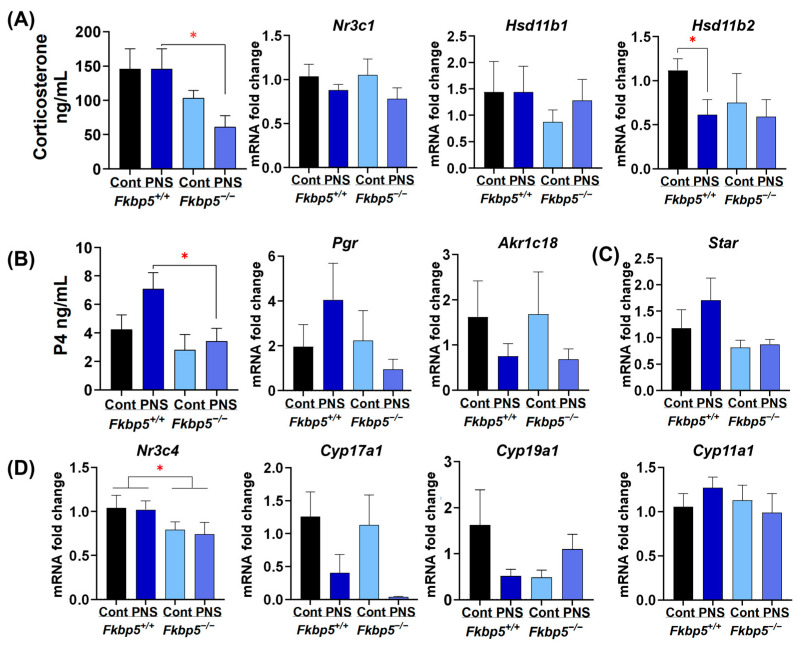
Evaluation of serum corticosterone and progesterone levels and ovarian expression profiles of target genes in adult offspring. (**A**) Serum corticosterone levels (*n* = 6/group) as well as the expression of glucocorticoid receptor (*Nr3c1*) and 11β-hydroxysteroid dehydrogenase (*Hsd11b1* and *b2*) genes in the ovary. (**B**) Serum progesterone (P4) levels (*n* = 6/group) as well as expression of progesterone receptor (*Pgr*) and aldo-keto reductase family 1-member c18 (*Akr1c18*) mRNA levels in the ovary; (**C**) expression of steroidogenic enzymes steroidogenic acute regulatory protein (*Star*) and Cytochrome P450 family 11, subfamily a, polypeptide 1 (*Cyp11a1*); and (**D**) androgen receptor (*Nr3c4*), Cytochrome P450 family 17, subfamily a, polypeptide 1 (*Cyp17a1*) and family 19, subfamily a, polypeptide 1 (*Cyp19a1*) levels in the ovary. Bars represent mean ± SEM, *Hsd11b2 Fkbp5*^+/+^ PNS *n* = 7, the rest *n* = 6; * *p* < 0.05.

We previously reported that [17] restraint stress significantly elevates serum corticosterone levels in pregnant *Fkbp5*^+/+^ mice, whereas pregnant *Fkbp5*^−/−^ mice display significantly lower serum corticosterone levels in response, indicating that *Fkbp5*^−/−^ mice are resistant to stress-induced changes. Therefore, we first measured serum levels of corticosterone as a main glucocorticoid involved in regulation of stress in the mouse [52]. In adult offspring, PNS administration did not induce an increase in serum corticosterone levels in either *Fkbp5*^+/+^ (123.3 ± 15.7 ng/mL) or *Fkbp5*^−/−^ (64.6 ± 10.6) mice compared to their respective control (131.6 ± 18.8 and 93.4 ± 12.5, respectively). However, multiple factor ANOVA indicated that serum corticosterone levels were lower in both control and PNS-induced *Fkbp5*^−/−^ mice compared to wild-type mice (*p* < 0.01). This reduction was significantly lower in PNS-induced *Fkbp5*^−/−^ versus PNS-induced *Fkbp5*^+/+^ mice (64.6 ± 10.6 versus 123.3 ± 15.7; *p* < 0.05, Figure 3A), although it did not attain significance in control *Fkbp5*^−/−^ (93.4 ± 12.5) versus control *Fkbp5*^+/+^ (131.6 ± 18.8) mice (*p* = 0.1, Figure 3A). We also measured the levels of *Nr3c1* gene encoding GR [53]. However, ovarian *Nr3c1* levels did not differ among groups (Figure 3A).

The local effects of glucocorticoids are not solely dependent on serum concentrations, since intracellular glucocorticoid levels are tightly regulated by two isoforms of 11β-HSD. The *HSB11β1* converts inactive corticosterone to active form, while *HSD11β2* reverses this reaction, producing the inactive form of glucocorticoids at tissue levels [7]. Thus, to investigate the impact of PNS on local glucocorticoid regulation within the ovary, we measured the relative expression of ovarian *Hsd11b1* and *Hsd11b2* mRNA levels. Levels of *Hsd11b1* did not differ among groups in adult mice (Figure 3A), whereas *Hsd11b2* levels were significantly lower in PNS-induced *Fkbp5*^+/+^ mice (0.6 ± 0.2) versus controls (1.1 ± 0.1; *p* < 0.05, Figure 3A), but not in PNS-induced *Fkbp5*^−/−^ mice (0.6 ± 0.2) versus control *Fkbp5*^−/−^ (0.7 ± 0.3; *p* = 0.9) mice, suggesting that reduced *Hsd11b2* levels contribute to increased local availability of glucocorticoids in PNS-induced *Fkbp5*^+/+^ mice.

### 2.4. PNS Effects on Progesterone Signaling in Adult Offspring

The stress effect on P4 and its metabolism has been reported as either stimulatory [54] or inhibitory [55,56] in the literature [57]. Thus, we first investigated the impact of PNS on serum progesterone levels. Serum progesterone levels were similar between control *Fkbp5*^+/+^ (4.2 ± 1.0) and *Fkbp5*^−/−^ (2.8 ± 1.1; *p* = 0.3) adult mice. However, PNS administration significantly increased serum progesterone levels in *Fkbp5*^+/+^ (7.1 ± 1.1) versus *Fkbp5*^−/−^ mice (3.3 ± 0.9; *p* < 0.05, Figure 3B).

Later, we measured ovarian expression of *Pgr*, which encodes PR, which plays a crucial role in regulating ovulation and luteinization [58]. We also measured the levels of the aldo-keto reductase gene *Akr1c18* encoding 20α-hydroxysteroid dehydrogenase enzyme, which metabolizes P4 into the less potent 20α-hydroxyprogesterone [59]. Analysis of qPCR results revealed that (1) in adult mice, *Pgr* relative expression did not significantly differ in *Fkbp5*^+/+^ PNS mice (3.7 ± 1.4) compared to *Fkbp5*^−/−^ PNS mice (0.9 ± 0.4; *p* = 0.2, Figure 3B) and (2) the relative expression of *Akr1c18* was not significantly different in both PNS-induced *Fkbp5*^+/+^ (0.7 ± 0.2) and *Fkbp5*^−/−^ (0.6 ± 0.2) mice compared to their corresponding controls (1.5 ± 0.6 and 1.5 ± 0.8, respectively; *p* = 0.6, Figure 3B).

Ovarian steroidogenesis is the process through which ovarian cells produce hormones for maintaining reproductive tissues and regulating ovarian function [60]. To investigate if PNS caused any changes in steroidogenesis regulation, we assessed mRNA levels of (1) *Star*, the first enzyme responsible for transporting cholesterol into the inner mitochondrial membrane [61], and (2) *Cyp11a1*, which converts imported cholesterol to pregnenolone [61]. However, ovarian levels of *Star* and *Cyp11a1* levels did not differ among groups (Figure 3C).

### 2.5. The Impact of PNS on Androgen Signaling in Adult Offspring

Androgens, via binding to the androgen receptor encoded by the *Nr3c4* gene, are essential for maintaining ovarian function, i.e., follicular growth and estrogen synthesis, and they are also implicated in ovarian disease such as polycystic ovarian syndrome [62]. In contrast to its effects on PR- and GR-mediated transcription, FKBP51 induces androgen receptor-mediated transcriptional activity [63]. Thus, to investigate the effects of PNS or genotype, we first measured the whole ovary expression of *Nr3c4*. In adult mice, multiple factor ANOVA analysis revealed a significant effect of genotype on relative *Nr3c4* mRNA expression (*p* < 0.05). Relative expression was lower in control (0.8 ± 0.1) and PNS-induced *Fkbp5*^−/−^ (0.7 ± 0.1) mice compared to their *Fkbp5*^+/+^ counterparts (1.0 ± 0.1 and 1.0 ± 0.1, respectively; Figure 3D).

Next, to investigate the effect of PNS on ovarian androgen synthesis, we measured ovarian expression of *Cyp17a1*, which catalyzes a first step in androgen synthesis from P4 metabolites [64]. In adult mice, PNS administration did not significantly decrease *Cyp17a1* expression in *Fkbp5*^+/+^ and *Fkbp5*^−/−^ mice compared to their own controls (0.4 ± 0.2 versus 1.3 ± 0.4, *p* = 0.051, and 0.04 ± 0.01 versus 1.1 ± 0.4, *p* = 0.06, respectively; Figure 3D). Later, we measured levels of *Cyp19a1* encoding the aromatase enzyme, which converts androgen precursors produced in the ovary to estrogens [65]. No significant change was detected in *Cyp19a1* relative expression in PNS-induced *Fkbp5*^+/+^ mice (0.4 ± 0.1) compared to control *Fkbp5*^+/+^ mice (1.6 ± 0.6; *p* = 0.3, Figure 3D).

### 2.6. PNS Administration Increases Primordial Follicle Atresia in Peripubertal Fkbp5^+/+^ Offspring

**Figure 4:**
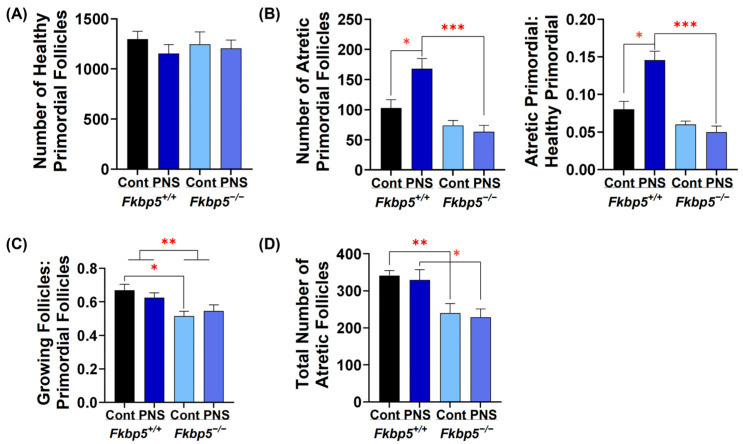
PNS-induced atresia in peripubertal offspring. (**A**) The number of healthy primordial follicles in peripubertal control or PNS *Fkbp5*^+/+^ and *Fkbp5*^−/−^ offspring; (**B**) the atretic primordial follicles and the ratio of atretic to healthy primordial follicles indicating the loss of primordial follicles; (**C**) the ratio of total healthy growing to healthy primordial follicles; and (**D**) total number of atretic growing follicles. Bars represent mean ± SEM, *n* = 6/each, * *p* < 0.05; ** *p* < 0.01; *** *p* < 0.001.

To determine if the reduction in ovarian reserve observed in PNS *Fkbp5*^+/+^ adult mice was due to lower initial ovarian reserve, we counted the number of follicles in ovaries collected from peripubertal mice at 24 days. The number of healthy primordial follicles was similar in either PNS-induced *Fkbp5*^+/+^ (1154.6 ± 88.6) or PNS-induced *Fkbp5*^−/−^ (1205.8 ± 81.4) mice compared to their controls (1298.3 ± 77.3 and 1245.8 ± 124.4, respectively; Figure 4A). However, a significantly higher number of atretic primordial follicles was found in PNS-induced *Fkbp5*^+/+^ mice (167.9 ± 16.9) versus *Fkbp5*^+/+^ controls (102.9 ± 13.9; *p* < 0.05, Figure 4B), but not in PNS-induced *Fkbp5*^−/−^ versus *Fkbp5*^−/−^ controls. The number of atretic primordial follicles was also significantly higher in PNS-induced *Fkbp5*^+/+^ mice (167.9 ± 16.9) versus PNS-induced *Fkbp5*^−/−^ mice (63.3 ± 10.7; *p* < 0.001, Figure 4B). We then calculated the ratio of atretic primordial follicles to healthy primordial follicles to assess the extent of primordial follicle activation leading to atresia as opposed to growth. In peripubertal mice, PNS significantly increased this ratio in *Fkbp5*^+/+^ compared to *Fkbp5*^+/+^ control or *Fkbp5*^−/−^ PNS mice (0.14 ± 0.01 versus 0.08 ± 0.01, *p* < 0.05, or versus 0.05 ± 0.01, *p* < 0.001, respectively; Figure 4B). However, PNS administration did not alter the ratio in *Fkbp5*^−/−^ mice (Figure 4B), indicating that *Fkbp5* deletion prevents ovarian follicular atresia induced by PNS in mice.

### 2.7. Fkbp5 Depletion Reduces Follicle Recruitment in Peripubertal Control Offspring

To investigate if PNS affects follicle recruitment, the process of activating dormant primordial follicles into the growing follicle pool [66], we counted the numbers of growing follicles including primary, secondary, tertiary, and early antral follicles. We then calculated the ratio of growing to primordial follicles to determine the rate of follicle recruitment among groups. PNS administration did not alter the rate of follicle recruitment in either *Fkbp5*^+/+^ (0.62 ± 0.03 versus 0.67 ± 0.03) or *Fkbp5*^−/−^ (0.55 ± 0.04 versus 0.51 ± 0.03, Figure 4C) mice, indicating no additional PNS effect. However, the recruitment rate was significantly lower in *Fkbp5*^−/−^ than *Fkbp5*^+/+^ mice (F [1, 20] = 12.686; *p* < 0.01, Figure 4C), suggesting a regulatory mechanism that controls the transition from primordial to growing follicles in *Fkbp5*^−/−^ mice. Additionally, significantly fewer atretic follicles were observed in *Fkbp5*^−/−^ versus *Fkbp5*^+/+^ control (240 ± 25.3 versus 341.3 ± 13.7; *p* < 0.01) and in *Fkbp5*^−/−^ versus *Fkbp5*^+/+^ PNS mice (228.8 ± 22 versus 329.6 ± 27.1; *p* < 0.05, Figure 4D). However, PNS administration did not alter the total number of atretic follicles in *Fkbp5*^+/+^ and *Fkbp5*^−/−^ mice compared to their own controls (*p* = 0.7 and *p* = 0.7, respectively, Figure 4D), indicating that the effects are genotype-specific rather than due to PNS administration.

### 2.8. PNS Delays Pubertal Onset in Fkbp5^+/+^ Mice

In mice, vaginal opening is widely accepted as a marker for the onset of puberty [67]. To understand pubertal maturation, we monitored vaginal opening (Figure S2) in peripubertal mice across groups. No significant difference was observed between control Fkbp5^+/+^ versus Fkbp5^−/−^ mice (*p* = 0.1). However, PNS administration significantly delayed vaginal opening in PNS-induced Fkbp5^+/+^ versus Fkbp5^+/+^ control mice (32.63 *±* 0.9 versus 29.2 *±* 0.5, respectively, *p* < 0.05; Table 1), but not in PNS-induced Fkbp5^−/−^ (33.33 *±* 0.96) versus Fkbp5^−/−^ control mice (31.08 *±* 0.8, *p* = 0.1).

### 2.9. PNS Causes a Rapid Decline in Ovarian Reserve in Middle-Aged Fkbp5^+/+^ Mice

**Figure 5:**
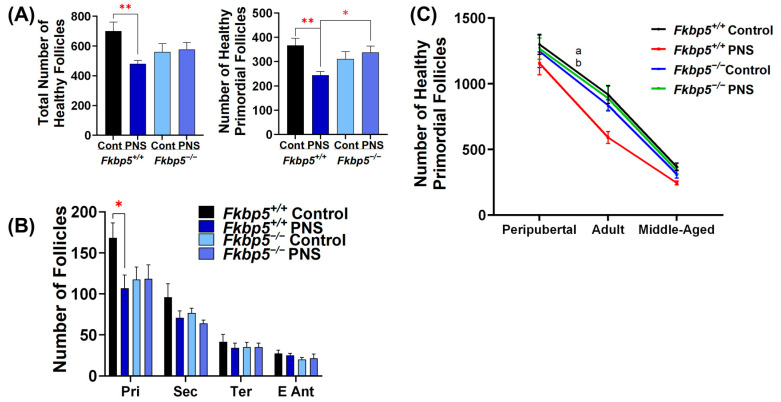
Reproductive aging is associated with a progressive decline of ovarian reserve in middle-aged *Fkbp5*^+/+^ PNS offspring. (**A**) Reduced total healthy follicle numbers (480.8 ± 22.5 versus 700 ± 59.5; *p* < 0.01) and healthy primordial follicle numbers in middle-aged *Fkbp5*^+/+^ PNS mice versus *Fkbp5*^+/+^ control mice (244.2 ± 15 versus 366.7 ± 28.6, *p* < 0.01); (**B**) the number of growing follicles, including primary, secondary, tertiary, and early antral follicles, among middle-aged groups. Reduced primary follicle number in middle-aged *Fkbp5*^+/+^ PNS versus middle-aged *Fkbp5*^+/+^ control mice (106.7 ± 16.2 versus 168.3 ± 18.2; *p* < 0.05); (**C**) the dynamic changes in number of healthy primordial follicles from peripubertal to adult and then to middle-aged mice. The number of primordial follicles rapidly declined in *Fkbp5*^+/+^ PNS versus *Fkbp5*^+/+^ control (independent-samples *t*-test; a *p* < 0.01) and *Fkbp5*^−/−^ PNS (independent-samples *t*-test; b *p* < 0.05) groups between peripubertal and adult age points. Bars represent mean ± SEM, *n* = 6/each, * *p* < 0.05; ** *p* < 0.01.

Reproductive aging involves a natural, progressive decline in follicle number, leading to fertility loss due to ovarian dysfunction [68,69]. To define whether PNS accelerates this decline with aging, we investigated the total follicle number in middle-aged (10-month-old) mice. As shown in adult offspring (Figure 1B), in middle-aged groups, we found a significantly reduced number of total healthy follicles in PNS-induced *Fkbp5*^+/+^ (480.8 ± 22.5) versus *Fkbp5*^+/+^ control mice (700 ± 59.5; *p* < 0.01; Figure 5A), but this effect was not observed in PNS-induced *Fkbp5*^−/−^ PNS (577.5 ± 46.7) versus *Fkbp5*^−/−^ control mice (560 ± 54.6; *p* = 0.8, Figure 5A). Moreover, the number of healthy primordial follicles was significantly lower in PNS-induced *Fkbp5*^+/+^ (244.2 ± 15.0) versus *Fkbp5*^+/+^ control mice (366.7 ± 28.6, *p* < 0.01) or PNS-induced *Fkbp5*^−/−^ (338.3 ± 25.5; *p* < 0.05, Figure 5A), suggesting that PNS exacerbates the progressive loss of ovarian reserve in *Fkbp5*^+/+^ mice as they age. We then counted the growing follicles. In middle-aged mice, the number of healthy primary follicles was significantly lower in PNS-induced *Fkbp5*^+/+^ (106.7 ± 16.2) versus *Fkbp5*^+/+^ control mice (168.3 ± 18.2; *p* < 0.05), but not in PNS-induced *Fkbp5*^−/−^ versus *Fkbp5*^−/−^ control mice (118.3 ± 17 versus 117.5 ± 15.3; *p* = 1.0, Figure 5B). However, the number of healthy secondary, tertiary, and antral follicles did not differ among groups (Figure 5B).

To better understand the changes in the ovarian reserve across the reproductive life course, we assessed the percentage change in healthy primordial follicles between the peripubertal and adult age groups and between the adult and middle-aged groups. The percentage change in healthy primordial follicles from the peripubertal to the adult was significantly greater in PNS-induced Fkbp5^+/+^ (−48.7% ± 2.5) than in Fkbp5^+/+^ control mice (−28.9% ± 4.84; *p* < 0.01) or in PNS-induced Fkbp5^−/−^ mice (−28.9% ± 7.5; *p* < 0.05, Figure 5C). Conversely, PNS administration did not alter the percent change in healthy primordial follicles from adult to middle age in Fkbp5^+/+^ (−56.7% ± 5.4) and Fkbp5^−/−^ (−59% ± 6.3) mice compared to their respective controls (−59.2% ± 3.9 and −62.2% ± 4, respectively).

### 2.10. The Effect of PNS on FKBP51 Expression in the Middle-Aged Offspring Ovary

**Figure 6:**
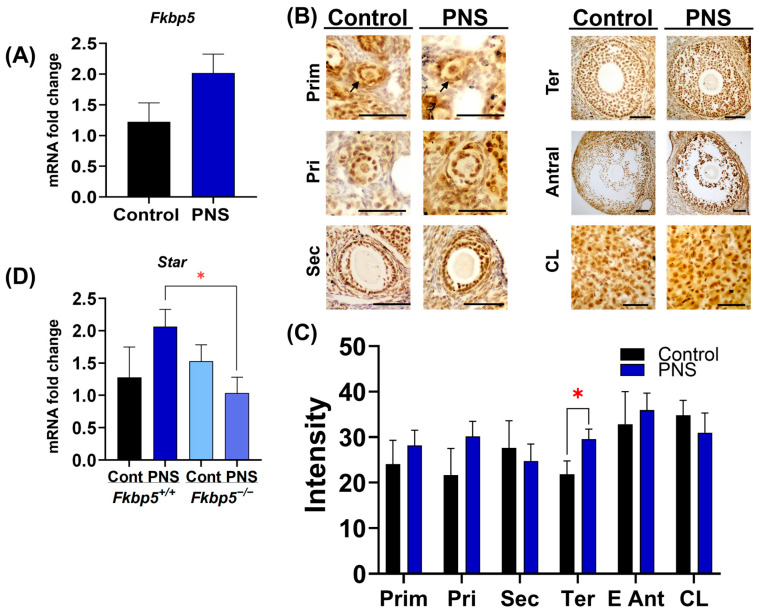
Expression of FKBP51 in middle-aged offspring ovaries. (**A**) Ovarian *Fkbp5* expression by qPCR from middle-aged *Fkbp5*^+/+^ control and PNS mice. Bar represents mean ± SEM, *n* = 6/each. (**B**) Representative FKBP51 immunoreactivity (brown) in granulosa and luteal cells from 10-month-old *Fkbp5*^+/+^ control and PNS mice. Scale bars = 20 µm. (**C**) Bars show intensity of FKBP51 immunostaining in every follicular stage and corpus luteum. Prim: primordial; Pri: primary; Sec: secondary; Ter: tertiary; E Ant: early antral; and CL: corpus luteum. Bars represent mean ± SEM, *n* = 6 mice/group; compared to PNS versus control, primordial (Prim; 28.2 ± 3.3 versus 24.1 ± 5.2; *p* = 0.5); primary (Pri; 30.2 ± 3.3 versus 21.6 ± 5.8; *p* = 0.15); secondary (Sec; 24.7 ± 3.7 versus 27.7 ± 5.9; *p* = 0.8); tertiary (Ter; 21.9 ± 2.9 versus 29.5 ± 2.2; *p* < 0.05); early antral (E Ant; 36 ± 3.7 versus 32.9 ± 7.2; *p* = 0.16); corpus luteum (CL 31 ± 4.3 versus 34.8 ± 3.3; *p* = 0.2). (**D**) Ovarian *Star* expression by qPCR from middle-aged mice. Bars represent mean ± SEM, *n* = 6/each, * *p* < 0.05.

To investigate whether reproductive aging increases ovarian FKBP51 levels in middle-aged PNS *Fkbp5*^+/+^ mice, we performed qPCR and immunohistochemistry. PNS administration did not significantly increase *Fkbp5* relative expression (2.0 ± 0.3) compared to controls (1.2 ± 0.3; *p* = 0.1, Figure 6A). Immunostaining intensity of FKBP51 was comparable between groups in primordial, primary, secondary, and antral follicles (*p* ≥ 0.05, Figure 6B,C). However, in tertiary follicles, FKBP51 immunoreactivity was significantly higher in *Fkbp5*^+/+^ PNS mice compared to controls (29.5 ± 2.2 versus 21.9 ± 2.9; *p* < 0.05, Figure 6B,C).

### 2.11. PNS Disruptions in Cholesterol Transport in Middle-Aged Fkbp5^+/+^ Offspring Ovaries

Like adult mice, PNS administration did not significantly increase *Star* relative expression in middle-aged *Fkbp5*^+/+^ mice compared to controls (2.1 ± 0.5 and 1.3 ± 0.3, respectively; *p* = 0.2, Figure 6C). However, *Star* levels were significantly decreased in PNS-induced *Fkbp5*^−/−^ versus PNS-induced *Fkbp5*^+/+^ mice (1.5 ± 0.2; *p* < 0.05).

## 3. Discussion

The negative impact of maternal stress on pregnancy outcomes [70] and on the neurodevelopment of offspring [71] has been well documented. Additionally, both rat and human studies have demonstrated the adverse effects of early life events on female reproductive function [72,73,74], although there is variability between and limitations among these studies. Differences among stress models include variations in species, types of stressors, timing and duration of stress administration, age, and gender [75,76]. Therefore, in the current study, to assess the effects of PNS on reproductive function, we employed a maternal restraint stress model, which is accepted as a physiologic model designed to mimic everyday human stress, such as the repetitive stress of a demanding job or familial pressures [77]. This stress model was tested using *Fkbp5*^+/+^ and *Fkbp5*^−/−^ mice since previous results [78,79,80,81] clearly indicate that *Fkbp5*^−/−^ mice displayed maximal resistance to stress-related pathophysiologic changes. Thus, to evaluate the role of FKBP51 as a stress response protein in ovarian folliculogenesis across the lifespan, we selected *Fkbp5*^+/+^ and *Fkbp5*^−/−^ mice at three stages: 24 days (prepubertal), 2 months (reproductively active), and 10 months (reproductive aging). The period from 2 to 10 months represents a time of significant ovarian reserve decline, which is a hallmark of reproductive aging [82,83]. Thus, this is the first study to reveal the effects of PNS on ovarian function in both *Fkbp5*^+/+^ and *Fkbp5*^−/−^ mice and age-dependent ovarian FKBP51 expression in *Fkbp5*^+/+^ mice, as well as to demonstrate tolerance of *Fkbp5*^−/−^ mice to the PNS-induced decline in ovarian reserve.

Previous studies in humans, mice, and sparrows showed that stressful circumstances upregulate FKBP51 expression by activating the HPA axis and increasing glucocorticoid release [34,36,37,84]. Several studies have linked higher FKBP51 expression with increased susceptibility to post-traumatic stress disorders, major depressive disorders, and anxiety, as well as aging [85,86]. The current study demonstrates that maternal stress increased offspring FKBP51 levels primarily in granulosa cells of primary, secondary, and tertiary follicles in 2-month-old mice and in tertiary follicles in 10-month-old mice, while total ovarian *Fkbp5* mRNA levels were not altered. This suggests that the stress response may vary across different follicle stages, impact granulosa cell function, and play a critical role in the transition from growing preantral follicles to antral follicles preparing for ovulation. Such a mechanism could be crucial for women’s reproductive health, as defects in folliculogenesis are among the primary causes of female infertility [87].

Ovarian reserve is a key determinant of reproductive lifespan, as females are born with a finite number of oocytes, which gradually decreases over their lifetime [68,69]. Both stress and aging are well-known potential contributing factors to the decline of the ovarian reserve throughout the reproductive life [88,89]. Poulain et al. [28] previously demonstrated that exposure to glucocorticoid impaired fetal oogenesis by increasing apoptosis of germ cells in an in vitro cultured human fetal ovary, but this detrimental effect was observed in only high doses (10 and 50 µM, but not in 2 µM) of glucocorticoid. Thus, we counted primordial follicle number in the peripubertal ovaries to investigate whether the reduced primordial follicle numbers in 2-month-old *Fkbp5*^+/+^ PNS mice are associated with a lower ovarian reserve at birth due to higher corticosterone exposure during pregnancy, as indicated by our previous study [17], which found significantly higher serum corticosterone levels in PNS-induced *Fkbp5*^+/+^ dams versus *Fkbp5*^+/+^ controls and lower levels in PNS-induced *Fkbp5*^−/−^ and control *Fkbp5*^−/−^ dams.

However, in the peripubertal ovaries, the number of healthy primordial follicles was similar among groups. This could be explained by the possibility that the increased corticosterone levels in dams are not high enough to induce germ cell apoptosis, as suggested by Poulain et al. [28], or that the fetal ovary could be resistant to changes in GR signaling, as suggested by Cincotta et al. [90]. However, PNS-exposed *Fkbp5*^+/+^ mice in the peripubertal period showed increased primordial follicle atresia. This increased atresia may result from defective follicular activation, which triggers granulosa cell death during the transition into adulthood [91]. Supporting this, Liew et al. [92] showed the apoptotic mechanism underlying loss of primordial follicles during the pubertal window in mice (20–50 days) using pro-apoptotic protein BLC-2 modifying factor knocked out mice. Since no difference was found for atretic follicular numbers between PNS-induced *Fkbp5*^−/−^ and *Fkbp5*^−/−^ control mice, collectively, these results indicate prevention of PNS-associated follicular atresia in *Fkbp5*-deficient mice, suggesting FKBP5 as a potential therapeutic target in women with diminished ovarian reserve.

To investigate the impact of aging on ovarian reserve, we calculated the decline rate of primordial follicles throughout life and found the most significant loss between peripubertal and adulthood in *Fkbp5*^+/+^ PNS mice, but not in *Fkbp5*^−/−^ PNS mice. This indicates a physiological wave of primordial follicle loss during puberty, followed by a more gradual decline after sexual maturity. These findings are consistent with other studies in humans and mice demonstrating significant decline in primordial follicles during pubertal development [92,93,94,95,96]. Taken together, our findings suggest that *Fkbp5* deficiency may have a protective effect on PNS-exacerbated loss of primordial follicles around puberty.

A previous study reported that chronic stress increases the activation rate of primordial follicles, leading to higher numbers of primary follicles, possibly due to increased corticotropin-releasing hormone signaling in mice. However, this increase in primary follicles was accompanied by a decrease in growing follicles [97]. Similarly, our results show that peripubertal *Fkbp5*^+/+^ mice had higher levels of primordial follicle activation leading to growth compared to *Fkbp5*^−/−^ mice. This effect was not influenced by PNS (Figure 4C), suggesting that genotype, rather than PNS, affects primordial follicle selection for growth. Contrary to prepubertal mice, in adult and middle-aged *Fkbp5*^+/+^ mice, PNS exposure resulted in lower numbers of primary follicles, although the activation rate remained unchanged. This reduction in primary follicles in PNS-induced *Fkbp5*^+/+^ mice is likely due to a reduced pool of primordial follicles resulting from increased loss during puberty transition. This finding supports the idea seen in mice and cattle that the activation of primordial follicles is proportional to the remaining pool of dormant primordial follicles [98,99]. Taken together, the lower primary follicles observed in PNS-induced *Fkbp5*^+/+^ in adulthood can be explained by proportional activation of the primordial follicle pool. Conversely, in *Fkbp5*^−/−^ control mice, the reduction in primary follicles in adulthood can be explained by reduced activation of primordial follicles. These findings may help us to explain why PNS-induced middle-aged *Fkbp5*^+/+^ mice approach ovarian senescence more rapidly than the other groups.

Middle-aged mice displayed significantly elevated ovarian levels of *Star* in *Fkbp5*^+/+^ PNS mice. Changes in *Star* expression in *Fkbp5*^+/+^ mice may be associated with activation of primordial follicles, since a recent study showed cholesterol metabolism mediated activation of primordial follicles in mice [100]. Additionally, increased *Star* expression was reported in theca cells of growing follicles in patients with polycystic ovarian syndrome, which is characterized by hyperandrogenism and abnormal follicle growth [101]. Since FSH and LH through the cAMP-PKA pathway are main regulators of *Star* expression [102], contribution of this pathway to mediating PNS-induced ovarian changes needs further investigation. Ding et al. [103] also demonstrated increased follicular atresia accompanied with abnormal upregulation of P4 and STAR levels in cold-exposed female mice. In this context, upon reaching sexual maturity, PNS-induced *Fkbp5*^+/+^ mice showed elevated FKBP51 levels in granulosa cells, which may reduce PR sensitivity and increase P4 EC_50_ for PR activation [31], explaining the higher serum P4 levels compared to PNS-induced *Fkbp5*^−/−^ mice.

Creutzberg et al. [76] revealed that a PNS-induced rise in glucocorticoid is more consistently seen in PNS models using rats but is not necessarily consistent in mouse models. In our model, PNS did not increase serum corticosterone levels in offspring. However, the depletion of *Fkbp51* did result in decreased serum corticosterone levels in adult female mice, as shown previously in mice [35,78]. In contrast to systemic corticosterone levels, the lower ovarian expression of *Hsd11b2* in PNS-induced *Fkbp5*^+/+^ mice but not in PNS-induced *Fkbp5*^−/−^ mice further supports the idea that PNS increases local corticosteroid effects by reducing *Hsd11b2* levels, suggesting that there is a *Fkbp5*-dependent local mechanism for governing the intra-follicular corticosteroid milieu, which may cause GR signaling overactivation.

Consistent with other studies in rats [22,23,104], *Fkbp5*^+/+^ PNS mice exhibited a significant delay in puberty, indicating potential irregularities in the hypothalamus. In humans, FKBP51 is associated with stress-induced psychiatric disorders [34], which commonly occur during puberty [105]. This effect may reflect dysregulation of the HPO/HPA axes, as the hormonal milieu changes dramatically throughout life, with puberty being a particularly critical period for the development of pathologies such as anxiety and depression [106].

A major limitation of our model is that the global knockout of *Fkbp5* makes it difficult to separate direct effects on the ovary from broader impacts on the hippocampus, hypothalamus, pituitary gland, and adrenal gland. In our previous study, we found that *Fkbp5*^+/+^ dams exposed to restraint stress exhibited the following: (1) significantly higher ovarian levels of *Ak1c18*, a progesterone-metabolizing enzyme, with no differences in levels of *Cyp11a1* and *Hsd3b2*, steroidogenic enzymes involved in progesterone production, compared to *Fkbp5*^+/+^ control dams or control and PNS-exposed *Fkbp5*^−/−^ dams. Importantly, there was no difference in corpus luteal morphology or regression; (2) shortened gestational length and induced preterm birth in *Fkbp5*^+/+^ dams, but not in *Fkbp5*^−/−^ dams, indicating greater tolerance to stress; and (3) increased maternal serum corticosterone levels in *Fkbp5*^+/+^ dams, but significantly lower levels in *Fkbp5*^−/−^ dams, which also experienced prolonged gestation [17]. In the current study, most of the effects observed in the offspring ovaries are due to prenatal exposure, while differences in the ratio of growing follicles to primordial follicles (Figure 4C) and the total number of atretic follicles (Figure 4D) are attributed to the absence of the *Fkbp5* gene. Collectively, the results from our previous and current studies suggest that the effects of maternal restraint stress on both dams and offspring are mitigated by *Fkbp5* deletion.

Additionally, translating our findings to humans is challenging because mice do not experience true menopause [106]. However, using the global knockout and a physiological stress model more accurately reflects human conditions. Additionally, more pronounced effects are likely to be observed in humanized mouse models carrying the FKBP5 risk allele of the single-nucleotide polymorphism rs1360780, which enhances responsiveness to glucocorticoid stimulation [107].

## 4. Materials and Methods

### 4.1. Animals

*Fkbp5*^+/+^ and *Fkbp5*^−/−^ mice were obtained by mating heterozygous breeders. *Fkbp5^+/−^* heterozygous breeders from a mixed SvJ × C57BL/6–129 background were generated by replacing the first coding exon 2 with a targeting vector containing the lacZ and PGK^neo^ cassette, as previously described [79,108]. Mice were maintained in a temperature and humidity-controlled room under a 12:12 h light/dark cycle, housed in ventilated cages, and they received ad libitum access to water and food. All experiments were performed in accordance with the Institutional Animal Care and Use Committee (IACUC), and the breeding and experimental procedures were approved by the IACUC at the University of South Florida (10898M, 10574R, respectively).

### 4.2. Experimental Design

Female *Fkbp5*^+/+^ and *Fkbp5*^−/−^ mice (8 to 12 weeks old) were mated with an adult *Fkbp5*^+/+^ and *Fkbp5*^−/−^ male (12 to 24 weeks old), respectively, by housing them together in a 1:1 ratio for 4 h. Pregnancy was confirmed by the presence of a vaginal plug, which was designated as gestational day 0. Time-mated pregnant dams were then randomly assigned to either the control or maternal restraint stress (prenatally stressed, PNS) group. PNS was administered daily for 1 h three times for 10 consecutive days using a standard restraint chamber (1.5″ × 4″; Braintree Scientific, Inc, Braintree MA, USA.), starting on gestational day (E) 8 through 18. The control group was left undisturbed during the whole pregnancy (Figure S3). To prevent habituation, the stress schedule varied each day. Female offspring from the four dam groups (*Fkbp5*^+/+^ control, *Fkbp5*^+/+^ PNS, *Fkbp5*^−/−^ control, and *Fkbp5*^−/−^ PNS) were used for experimental analyses at postnatal day 24 (peripubertal), 2 months (adult), and 10 months (middle-aged).

### 4.3. Determination of Estrous Cycle Phase

The estrous cycle of mature female offspring mice was assessed using vaginal cytology, as described [109]. The stages of the estrous cycle were identified based on the predominant cell types: nucleated epithelial cells (proestrus), cornified squamous epithelial cells (estrus), a mix of nucleated epithelial cells, squamous epithelial cells, and leukocytes (metestrus), and leukocytes (diestrus). Briefly, vaginal smears were collected using a Pasteur pipette with phosphate-buffered saline (PBS, pH: 7.0–7.4; ThermoFisher, Waltham, MA, USA). The suspension placed on a microscope slide was air-dried, stained with 0.1% crystal violet (ThermoFisher) for 1 min, and then washed with tap water to remove excess staining. The slides were examined under light microscopy at 200× magnification. Mice assigned to estrus were euthanized for tissue collection.

### 4.4. Sampling Procedure

Mice were deeply anesthetized with isoflurane anesthesia to collect blood samples via cardiac puncture and then euthanized by cervical dislocation to harvest ovarian tissues between 9:00 a.m. and 12:00 p.m. to minimize the effects of circadian rhythm on hormone levels. To obtain serum, blood samples were collected in lithium heparin separator tubes (BD; Franklin Lakes, NJ, USA), centrifuged for 10 min at 6000 rpm at room temperature, and stored at −80 °C until hormone quantification. Ovaries were dissociated from the surrounding fat and oviduct under dissection microscopy. The left ovary was stored at −80 °C for RNA analysis, whereas the right one was fixed in 4% paraformaldehyde (PFA) solution for 18 h at room temperature. Later, tissue was dehydrated in serial ethanol solutions and embedded in paraffin for immunohistochemistry analysis or follicle counting.

### 4.5. Histomorphometric Analysis of Folliculogenesis in Ovaries

Serial sections (4 μm thick) from PFA-fixed, paraffin-embedded ovaries obtained from control and PNS *Fkbp5*^+/+^ or *Fkbp5*^−/−^ mice at 24 days, 2 months, and 10 months (*n* = 6/group per age) were stained with Periodic acid-Schiff (PAS; Sigma-Aldrich; St. Louis, MO, USA) according to the manufacturer’s protocol and counterstained with hematoxylin. Every 5th section was analyzed, and the total number of follicles per ovary was determined by counting the follicles with visible oocyte-displaying nuclei, then multiplying the count by five. Primordial, primary, secondary, preantral (tertiary), and antral follicles were classified as previously described [110,111,112]. Briefly, primordial follicles were defined as a small oocyte surrounded by a layer of squamous granulosa cells; primary follicles were described as an enlarged oocyte surrounded by a single layer of cuboidal granulosa cells; secondary follicles possessed an oocyte surrounded by two layers of cuboidal granulosa cells, with the presence of a theca cell layer; tertiary follicles contained an oocyte surrounded by more than two layers of cuboidal granulosa cells without an antrum and surrounded by theca cells; and early antral follicles were defined as a large follicle containing several layers of granulosa cells with an antral cavity, as well as the presence of theca interna and externa layers [110]. Atretic follicles were characterized by a degenerating oocyte with pyknotic granulosa cells, a loss of organized follicular structure, and/or the presence of the zona pellucida remnants [111]. Follicles were examined under an Axio Imager II microscope (Zeiss; White Plains, NY, USA) using ZEN 2011 software.

### 4.6. Reverse Transcription and Quantitative Real Time (q)-PCR Analysis

Total RNA was extracted from the ovaries of control and PNS *Fkbp5*^+/+^ or *Fkbp5*^−/−^ mice at 2 months (*Hsd11b2 Fkbp5*^+/+^ PNS *n* = 7, the rest *n* = 6) and 10 months (*n* = 6/group) using the RNEasy Mini Kit (Qiagen, Valencia, CA, USA) according to the manufacturer’s protocol. After DNase treatment (Qiagen), 1 µg total RNA from each sample was reverse transcribed using the RETROscript kit (Ambion, Austin, TX, USA) with the following cycling conditions: 85 °C for 3 min, 42 °C for 1 h, and 92 °C for 10 min to inactive reverse transcriptase enzyme. The expression levels of target genes (Table S1) were analyzed by qPCR using TaqMan gene expression assay (Applied Biosystems, Foster City, CA, USA) on an ABI 7500 thermocycler instrument. Each sample was run in duplicate, and the average value was used. Gene expression levels were normalized to *Actb* (β-actin) gene as an internal control. The 2^−△△Ct.^(cycle threshold) method was used to calculate relative expression levels and reported as fold-change in gene expression between different groups.

### 4.7. Immunohistochemistry and Quantification of Staining Intensity Analysis

Immunostaining was performed on 4% PFA-fixed, paraffin-embedded sections of ovaries from age- and cycle-matched controls and PNS *Fkbp5*^+/+^ or *Fkbp5*^−/−^ mice at 2 months and 10 months (*n* = 6 animals/group per age), as previously described [17]. After deparaffinization and antigen retrieval using citrate buffer (pH: 6.0) or 0.025% Trypsin-EDTA (Life Technologies: Grand Island, NY, USA), endogenous peroxidase activity was quenched with 3% hydrogen peroxide. Following washing steps with Tris-buffered saline with 0.1% Tween 20 (TBS-T), slides were blocked with 5% normal goat or horse serum (Vector Labs; Newark, CA, USA) for 30 min, then with an Avidin–Biotin blocking kit for 30 min (Vector Labs). The primary antibodies (Table S2) were applied overnight in a humidified chamber at 4 °C. After washing steps, the slides were incubated with biotinylated secondary antibodies (Vector Labs) for 30 min, and then washed and incubated with streptavidin-conjugated peroxidase complex (Vector Labs) for 30 min. After several rinses with TBS-T, immunoreactivity was developed using diaminobenzidine (Vector Labs) as the chromogen, and sections were counterstained with hematoxylin. For each protein of interest, three sections approximately 40 µm apart were microscopically examined.

The intensity of FKBP51 immunostaining was semi-quantitatively evaluated by using Photoshop-based image analysis methods, as described [113,114,115]. Briefly, all images were captured on an Axio Imager II microscope (Zeiss) using ZEN 2011 software at 200× magnification and exposure set to 40 ms to ensure standardization of optical settings. DAB-stained and corresponding negative control images were uploaded to Adobe Photoshop 7.0 (Adobe Inc., San Jose, CA, USA), converted to grayscale by removing RBG color information, and then adjusted using the invert command (Figure S2). Luminosity was measured using the rectangular marquee tool in 54 pixels (px) by 54 px areas with the Histogram command, and mean value was calculated. For quantification, all follicles were measured as follows: one measurement for small structures (primordial and primary follicles), the average of two measurements for secondary follicles, and the average of three measurements for larger tertiary follicles, antral follicles, corpora lutea, and ovarian stroma. Measures were also taken from negatively stained slides (hematoxylin-stained ovarian tissues), and the mean luminosity of negative measurements was subtracted from the mean luminosity of positive samples to remove background signals.

Immunostaining of cleaved caspase-3 cells was scored by the percentage of positively stained cells for each follicular stage. Scoring was performed independently by two blind investigators, and the average score of both was used.

### 4.8. Hormone Assessment

Serum P4 (*n* = 6/group) and corticosterone levels (*n* = 6/group) were measured in 2-month-old mice using ELISA kits (BioVendor Group; Brno, Czech Republic, and R&D Systems, Minneapolis, MN, USA, respectively) on the Bio-Rad Microplate Reader (Bio-Rad Laboratories; Hercules, CA, USA) according to the manufacturers’ protocol. Final concentrations of P4 and corticosterone were derived from the standard curve and analyzed using Microplate Manager Software Version 6.3 (Bio-Rad Laboratories, Hercules, CA, USA).

### 4.9. Statistical Analysis

Statistical analyses were performed using SPSS (version 28; IBM, Armonk, NY, USA). Data normality was assessed with the Kolmogorov–Smirnov test. Normally distributed data were analyzed by independent sample *t*-test or multi-factor ANOVA followed by post-hoc comparisons for data that contained two independent variables. If the data failed normality, the Mann–Whitney U test was used. A *p*-value of less than 0.05 was considered statistically significant. GraphPad Prism version 9.0 (GraphPad, San Diego, CA, USA) for Windows was used for graphing.

## 5. Conclusions

Our findings demonstrate that PNS delayed puberty, reduced the follicular reserve primarily through the loss of primordial follicles during puberty, and dysregulated steroidogenesis in an age-dependent manner. However, deletion of *Fkbp5* protects ovarian function against these PNS-induced adverse effects. The mechanisms mediated by FKBP51 are likely multifaceted, affecting the global regulation of the HPA and/or HPO axes. Overall, our findings provide the first evidence that maternal stress exposure in utero causes *Fkbp5*-dependent impairment of ovarian function in offspring and support FKBP51 as a potential therapeutic target.

## Figures and Tables

**Table 1:** Day of vaginal opening (VO) in female offspring as an approximation of pubertal onset. PNS significantly delayed VO in *Fkbp5*^+/+^ mice, but not in *Fkbp5*^−/−^ mice.

Experimental Group	*n*	Day of VO Mean ± SEM
*Fkbp5*^+/+^ Control	15	29.20 ± 0.56 ^b^
*Fkbp5*^+/+^ PNS	8	32.63 ± 0.96 ^a^
*Fkbp5*^−/−^ Control	12	31.08 ± 0.80
*Fkbp5*^−/−^ PNS	12	33.33 ± 0.96

^a^ Significantly different (*p* < 0.05) from *Fkbp5*^+/+^ control mice. ^b^ Significantly different (*p* < 0.05) from *Fkbp5*^+/+^ PNS mice.

## Data Availability

The original contributions presented in the study are included in the article/Supplementary Material; further inquiries can be directed to the corresponding author.

## Supplementary Materials

The following supporting information can be downloaded at https://www.mdpi.com/article/10.3390/ijms26062471/s1.
