# Supplementary material for: Depletion of Fkbp5 Protects Against the Rapid Decline in Ovarian Reserve Induced by Prenatal Stress in Female Offspring of Wild-Type Mice
Source: Int J Mol Sci. 2025 Mar 10;26(6):2471. doi: 10.3390/ijms26062471 (PMC11942629; doi:10.3390/ijms26062471)
Supplement: Supplementary file 1 [file ijms-26-02471-s001.zip › ijms-3428431-supplementary.pdf]

Supplementary Materials

Depletion of *Fkbp5* Protects Against the Rapid Decline in Ovarian Reserve Induced by Prenatal Stress in Female Offspring of Wild-Type Mice

Monica Moore [1], Busra Cetinkaya-Un [1], Papri Sarkar [1], Umit A. Kayisli [1], Nihan Semerci-Gunay [1], Michael Teng [2], Charles J. Lockwood [1] and Ozlem Guzeloglu-Kayisli [1,*]

Table S1. Transcripts of interest and their probe IDs for real-time quantitative PCR.

Gene	Full name	TaqMan probe ID
Fkbp5	FKBP prolyl isomerase 5	Mm00487406_m1
Nr3c1	Nuclear receptor subfamily 3 group C member 1 (glucocorticoid receptor)	Mm00433832_m1
Pgr	Progesterone receptor	Mm00435628_m1
Nr3c4	Nuclear receptor subfamily 3, group C, member 4 (androgen receptor)	Mm00442688_m1
Actb	Actin beta	Mm00607939_s1
Star	Steroidogenic acute regulatory protein	Mm00441558_m1
Cyp11a1	Cytochrome P450, family 11, subfamily a, polypeptide 1	Mm00490735_m1
Cyp19a1	Cytochrome P450, family 19, subfamily a, polypeptide 1	Mm00484049_m1
Cyp17a1	Cytochrome P450, family 17, subfamily a, polypeptide 1	Mm00484040_m1
Hsd11β1	Hydroxysteroid 11-beta dehydrogenase 1	Mm00476182_m1
Hsd11β2	Hydroxysteroid 11-beta dehydrogenase 2	Mm01251104_m1
Akr1c18	Aldo-keto reductase family 1, member C18	Mm00506289_m1

Table S2. List of primary and secondary antibodies used for immunohistochemistry.

Antibody Name	Catalog Number and Company	Species	Dilution Used for IHC
Goat IgG	Vector Laboratories, I-5000	Goat	1:50,000
Fkbp51	R&D Systems, AF4094	Goat	1:2000
Rabbit IgG	Vector Laboratories, I-1000	Rabbit	1:250,000
Caspase-3	Cell Signaling, 96645	Rabbit	1:2000
Biotinylated goat anti-rabbit IgG (H+L)	Vector Laboratories, BA-1000	Goat	1:400
Biotinylated horse anti-goat IgG (H+L)	Vector Laboratories, BA-9500	Horse	1:400

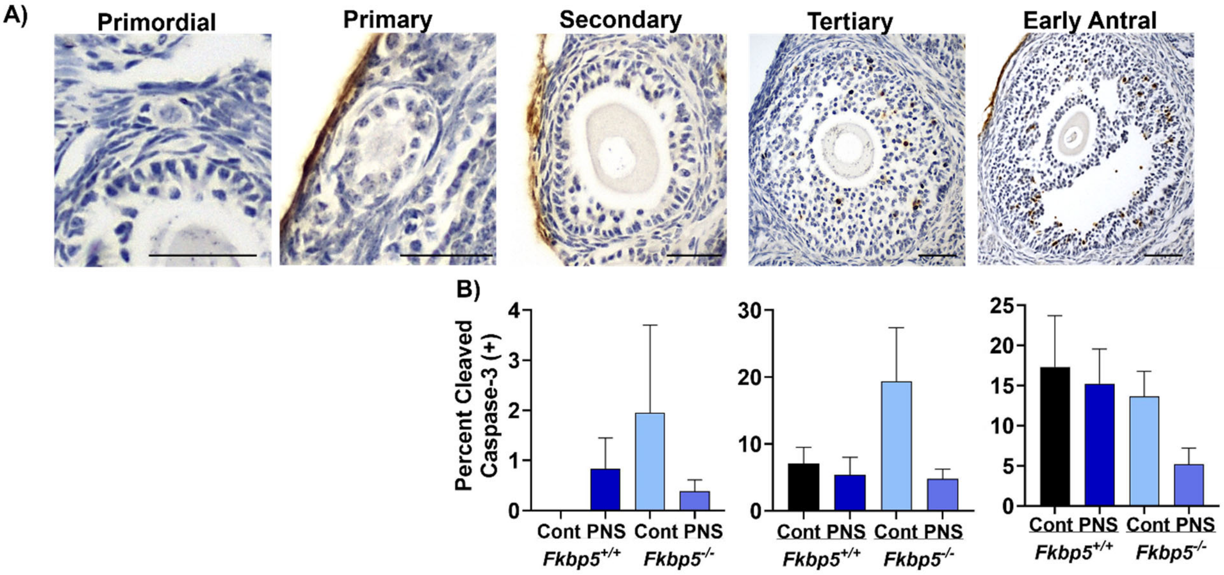

Supplementary Figure S1. Immunostaining of Caspase-3 in ovarian tissue of adult mice. (**A**) Representative images of active caspase-3 immunostaining from *Fkbp5*$^{+/+}$ control follicles at each follicular stage including primordial, primary, secondary, tertiary and early antral. Caspase 3 immunostaining is shown as brown color in granulosa cells of secondary, tertiary, and antral follicles; (**B**) Semi-quantitative evaluation of Caspase-3 immunostained granulosa cells in secondary follicles *Fkbp5*$^{+/+}$ control (0.0%± 0.0; n=25), *Fkbp5*$^{+/+}$ PNS (0.83%± 0.61; n=34), *Fkbp5*$^{-/-}$ control (1.95%± 1.75; n=20), and *Fkbp5*$^{-/-}$ PNS (0.39%± 0.22; n=36); tertiary follicles *Fkbp5*$^{+/+}$ control (7.0± 2.5; n=20), *Fkbp5*$^{+/+}$ PNS (5.4%± 2.6; n=19), *Fkbp5*$^{-/-}$ control (19.35%± 8.0; n=10), and *Fkbp5*$^{-/-}$ PNS (4.8%± 1.45; n=27); and early antral follicles *Fkbp5*$^{+/+}$ control (17.31%± 6.38; n=8), *Fkbp5*$^{+/+}$ PNS (15.24%± 4.32; n=19), *Fkbp5*$^{-/-}$ control (13.66%± 3.13; n=22) and *Fkbp5*$^{-/-}$ PNS (5.2%± 2; n=15). Scale bars=20 μm. Bars represent mean ± SEM.

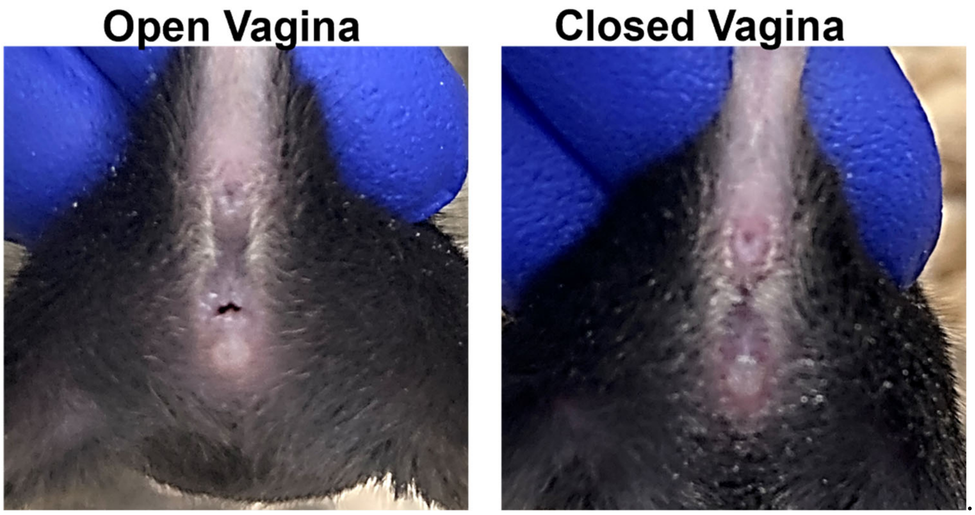

Supplementary Figure S2. Visualization of vaginal opening to estimate pubertal onset in female offspring. Representative photographs are shown to illustrate vaginal cavity in two states: an opened (left) and a closed (right) vaginal cavity to provide a visual reference for determining the timing of pubertal onset.

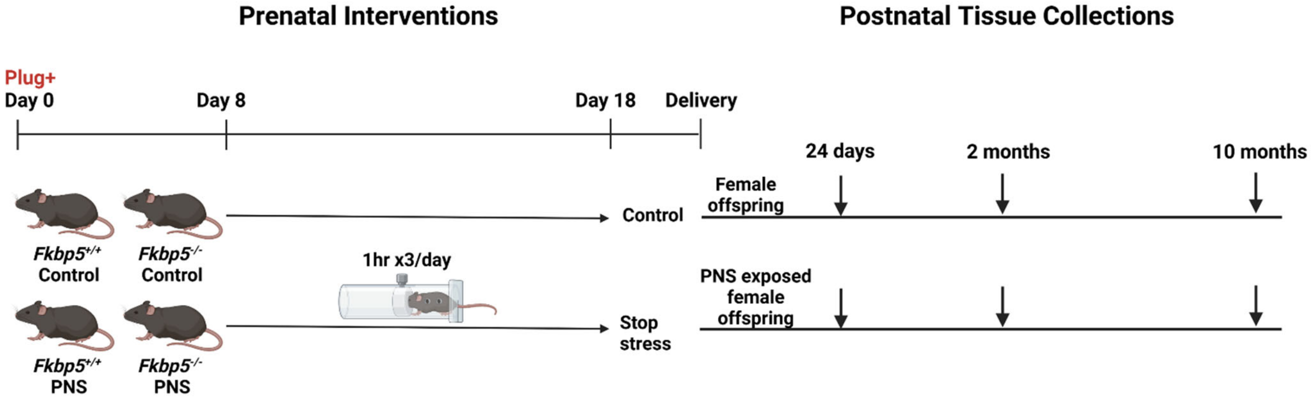

Supplementary Figure S3. Representative experimental design and methods. Schematic representation of the prenatal restraint stress (PNS) model and the timing of ovarian tissue sampling from female offspring. Time-mated pregnant dams were exposed to PNS staring to gestational day 8 through 18 using restraint chamber, while control dams were undistributed. Created in BioRender. Moore, M. (2025) https://BioRender.com/g50x813.

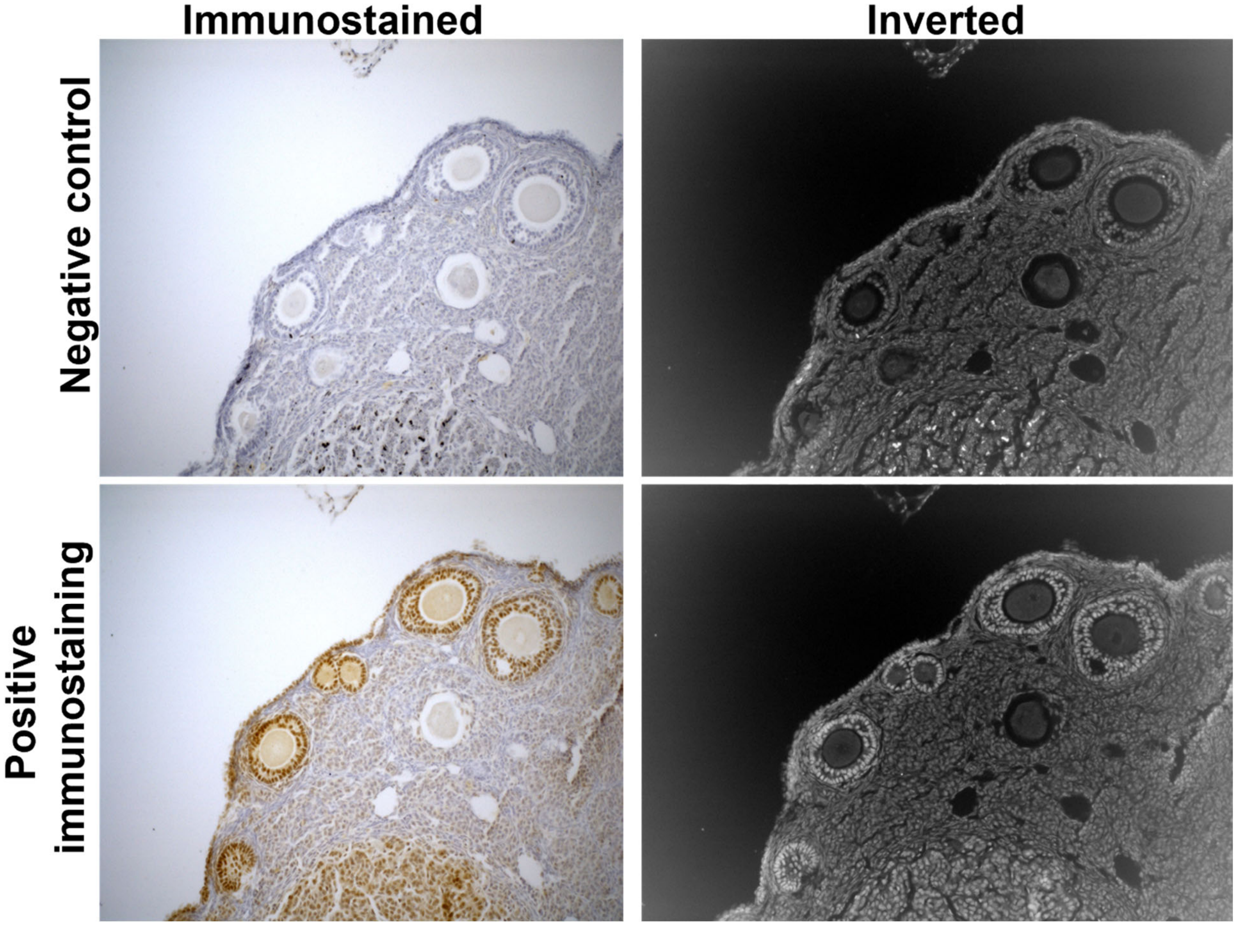

Supplementary Figure S4. Immunohistochemistry scoring for FKBP51 immunostaining in ovary. Image adjustments in Photoshop were made for FKBP51 immunostaining, including original H&E and FKBP51-stained images of serial sections. Grayscale and inverted images were used for analysis.
